# The joy of balancers

**DOI:** 10.1371/journal.pgen.1008421

**Published:** 2019-11-07

**Authors:** Danny E. Miller, Kevin R. Cook, R. Scott Hawley

**Affiliations:** 1 Department of Medicine, Division of Medical Genetics, University of Washington, Seattle, Washington, United States of America; 2 Department of Pediatrics, Division of Genetic Medicine, University of Washington, Seattle, Washington and Seattle Children's Hospital, Seattle, Washington, United States of America; 3 Department of Biology, Indiana University, Bloomington, Indiana, United States of America; 4 Stowers Institute for Medical Research, Kansas City, Missouri, United States of America; 5 Department of Molecular and Integrative Physiology, University of Kansas Medical Center, Kansas City, Kansas, United States of America; The University of North Carolina at Chapel Hill, UNITED STATES

## Abstract

Balancer chromosomes are multiply inverted and rearranged chromosomes that are widely used in *Drosophila* genetics. First described nearly 100 years ago, balancers are used extensively in stock maintenance and complex crosses. Recently, the complete molecular structures of several commonly used balancers were determined by whole-genome sequencing. This revealed a surprising amount of variation among balancers derived from a common progenitor, identified genes directly affected by inversion breakpoints, and cataloged mutations shared by balancers. These studies emphasized that it is important to choose the optimal balancer, because different inversions suppress meiotic recombination in different chromosomal regions. In this review, we provide a brief history of balancers in *Drosophila*, discuss how they are used today, and provide examples of unexpected recombination events involving balancers that can lead to stock breakdown.

The tools and techniques of the *Drosophila* genetics trade have evolved dramatically over the last century, but one instrument has stood the test of time—the balancer chromosome. Balancers are now an omnipresent and indispensable tool in the fly lab, and their importance has been recognized in other organisms as well. The multiple inversions and rearrangements that make up a balancer chromosome work to constrain recombination and impede the recovery of recombinant products. This allows for single deleterious alleles to be easily maintained in stock and also allows for the maintenance of mutations, transgenes, and/or chromosomal aberrations that are linked together in cis on the same chromosome. The presence of recessive lethal or sterile mutations on balancers assures that balancers never displace homologous chromosomes from stock populations while maintaining heterozygosity of deleterious mutations on the homologs. This combination of recombination suppression and enforced heterozygosity is what makes balancers so valuable to geneticists.

When a normal chromosome is combined with a balancer, the inversions prevent meiotic DNA double-strand breaks from being repaired as crossovers [1]. On those rare occasions that crossovers do form, balancers can prevent the recovery of recombinant chromosomes by one of two mechanisms, depending on the nature of the component inversion. A single exchange event within a paracentric inversion, which does not span the centromere, results in the formation of an acentric and a dicentric chromosome, neither of which will segregate properly during the subsequent meiotic divisions (Fig 1A). Single exchange events within pericentric inversions (those spanning the centromere) generate large deletions and duplications that are usually lethal to a developing embryo (Fig 1B). However, within an inverted segment, a double crossover between the same two chromatids does not lead to aneuploidy and, consequently, does not affect embryonic viability [2,3].

**Fig 1:**
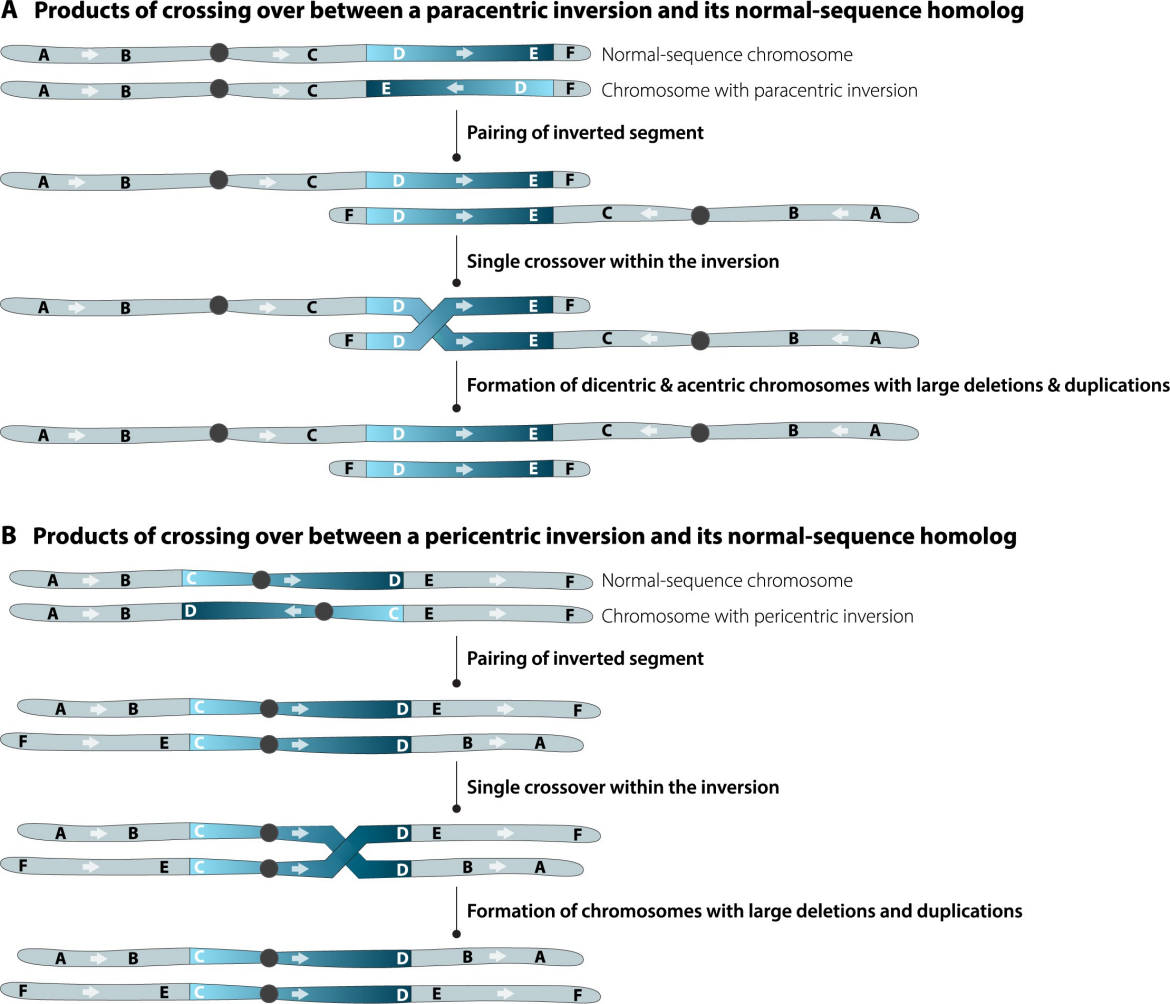
Common types of inversions. Paracentric inversions (A) do not encompass the centromere whereas pericentric inversions (B) do. Recombination between either type of inversion and a structurally wild-type homolog produces aneuploid chromosomes, which cause embryonic lethality.

## A brief history of balancer chromosomes

**Fig 2:**
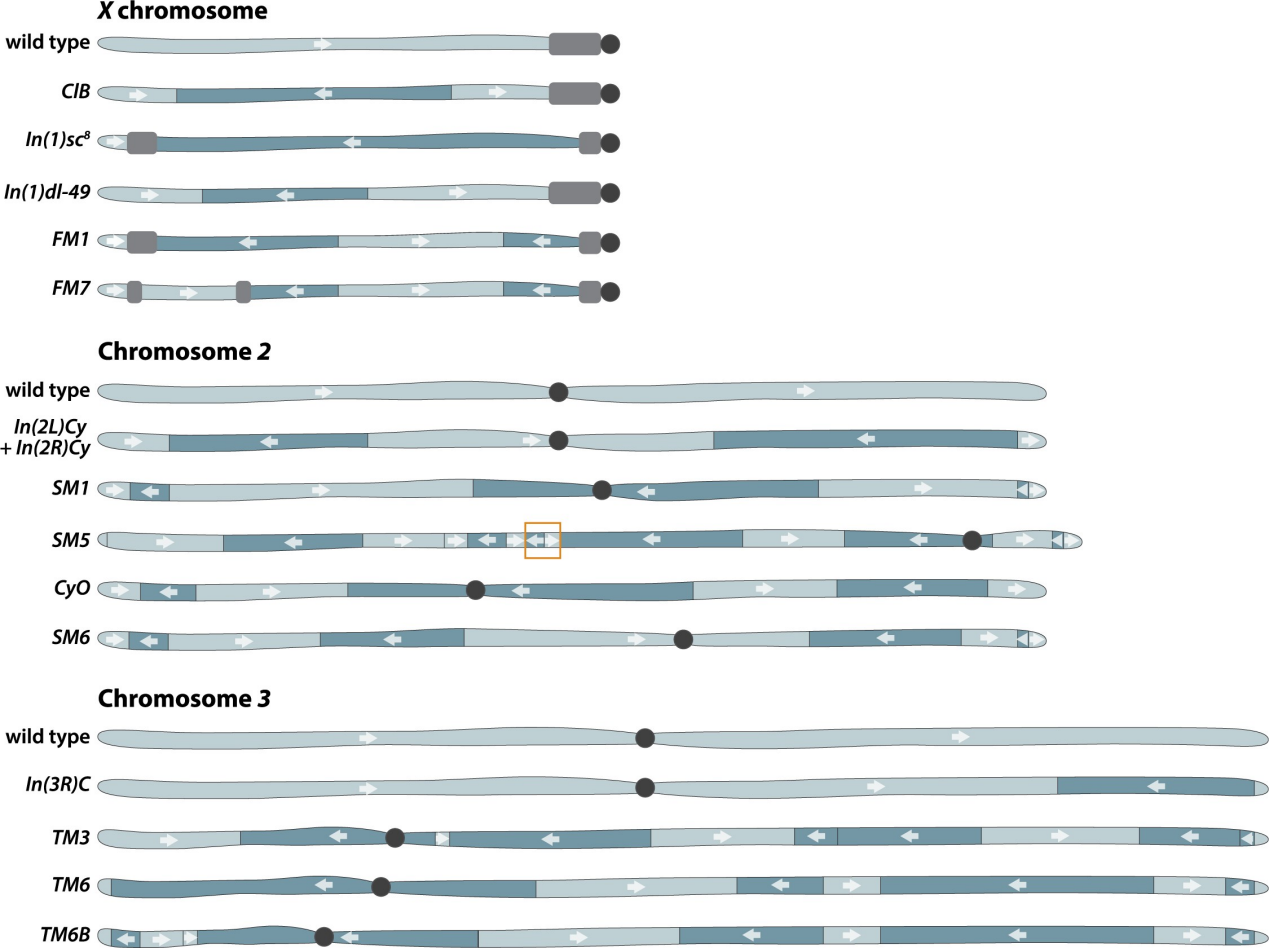
Commonly used balancer chromosomes in *Drosophila* showing approximate sizes of inverted segments. X chromosome balancers invert an uncertain amount of pericentric heterochromatin (gray). Second and third chromosome balancer inversions do not bisect centric heterochromatin, and thus heterochromatin is not represented here. The large duplication present on *SM5* is indicated (orange box).

The idea that heterozygosity for an inversion could suppress exchange was first proposed by Sturtevant [4] as a simple way to explain the observation that some chromosomes show a reduction of crossing over in a single region: “…individuals bearing one normal chromosome and one chromosome with an inverted section would probably show no crossing over in the region in question… .” As proof, he demonstrated that a crossover suppressor known as *C*_*III*_ [5] on Chromosome 3 was in fact an inverted segment later renamed *In(3R)C* [4,6]. This inversion, which involves the distal one-third of chromosome arm *3R*, is present on most third chromosome balancers used today (Fig 2).

Hermann Muller was likely the first to use the term “balanced” to describe enforced heterozygosity in a stock with a recessive lethal mutation on one chromosome and a crossover suppressor plus a recessive lethal on a homolog [7,8]. The first balanced stock involved *In(3R)C*, which carried a recessive lethal, in stock with a homolog carrying a recessive lethal allele of *Serrate* (*Ser)* with a dominant visible wing phenotype called *Beaded* (*Ser*^*Bd-1*^) [7]. Had there been free recombination between *In(3R)C* and the homolog, *Ser*^*+*^ from *In(3R)C* would have replaced *Ser*^*Bd-1*^, and the newly generated mutation-free chromosome would have quickly outcompeted both progenitor chromosomes to eliminate them from the stock population. By the 1920s, Muller had also characterized a large inversion of the middle of the X chromosome that carried several recessive visible markers, a dominant visible, and an unknown recessive lethal [9]. This chromosome, *ClB*, was an essential tool in the work that led to his Nobel prize in 1946. *In(1)dl-49*, a second X chromosome inversion discovered by Muller, inverts the middle third of the X, and although both chromosomes allowed infrequent proximal crossover events, *In(1)dl-49* did not allow frequent double crossovers inside of the inversion as *ClB* did. Additional X chromosome inversions were generated by Muller using X-irradiation, including several whole-arm inversions that placed centric heterochromatin near the distal *scute* gene [10].

Because it was obvious that single inversions did not fully suppress exchange over the entire X, efforts were undertaken to build a more effective balancer. By the 1950s, a balancer combining *In(1)dl-49* and a *scute* inversion existed and was known as *First Multiple 1* (*FM1)* [11]. With two inversions, this chromosome functioned well as a balancer [12]. Further X-irradiation resulted in additional inversions, which prevented the proximal exchanges that could occur on *FM1* [13,14]. One of the new balancers was *FM7*, the most commonly used X chromosome balancer today [15].

On Chromosome 2, Ward [16] was the first to describe two naturally occurring paracentric inversions that are the progenitors of nearly all second chromosome balancers used today. She was studying the *Curly* mutation isolated from a wild population and noted that when it was heterozygous, reductions in exchange occurred on both chromosome arms. Similar to the early X chromosome balancers, *In(2L)Cy + In(2R)Cy* was not an effective balancer for all second chromosome regions and was subjected to X-irradiation to introduce new inversions. A whole-chromosome inversion resulted in *Second Multiple 1* (*SM1)* [11], with subsequent irradiation of *SM1* resulting in *SM5*, which carried additional inversions and a large duplication [17]. Separate irradiation of *In(2L)Cy + In(2R)Cy* created a new balancer known as *Curly of Oster* (*CyO*) [18]. Single exchange events between *SM1* and *CyO* later produced *SM6* [19].

As noted above, third chromosome balancers also began as simple inversions that were combined and irradiated to produce more complex and effective balancers. X-irradiation of a third chromosome carrying two inversions, *In(3LR)sep* and *In(3R)C*, generated three new inversions to create *Third Multiple 3* (*TM3*) [20]. A different chromosome carrying three existing inversions, *In(3L)P*, *In(3LR)P88*, and *In(3R)C* was irradiated to create *TM6*; subsequent exchange of the left arm of *TM6* with another existing inversion, *In(3LR)HR33* (which also carried the three-breakpoint inversion *In(3R)Hu*), yielded *TM6B* [21–23].

The creation of balancers required a sophisticated understanding of chromosome manipulation and stands as a testament to the genius of midcentury *Drosophila* geneticists. In contrast, balancing mutations on the small fourth chromosome is simple. It does not undergo meiotic recombination and therefore does not need a multiply inverted chromosome to suppress exchange. Any fourth chromosome carrying a recessive lethal or sterile mutation can effectively act as a fourth chromosome balancer, although usually a recessive lethal mutation with a dominant visible phenotype, such as *eyeless*^*D*^, is used.

## Balancers in other species

Any chromosome carrying at least one inversion and a closely linked recessive lethal or sterile mutation can function as a balancer for specific chromosomal regions. Increasing the number of inversions and rearrangements allows a chromosome to function as a balancer for more regions, and dominant visible markers assist in following the balancer in crosses, but they are not absolutely necessary. Because crossovers are suppressed in the vicinity of any heterozygous aberration breakpoint, translocations, transpositions, and duplications can also be used as balancers—though these uses are rare in *Drosophila melanogaster*.

Inversions have been identified and studied in several *Drosophila* species in addition to *D*. *melanogaster*. For example, several inversions have been described on the third chromosome of *D*. *pseudoobscura*, including some that overlap [24]. Inversions have also been well described for many other species, and stocks with either inversions or translocations for at least *D*. *pseudoobscura*, *D*. *simulans*, and *D*. *virilis* are available at the National Drosophila Species Stock Center (http://blogs.cornell.edu/drosophila/).

Outside *Drosophila*, chromosomes acting as balancers can be found in a handful of organisms. For example, in *Caenorhabditis elegans*, chromosomes carrying translocations, duplications, or inversions function as balancers [25–30]. Together, these chromosomes cover most, if not all, of the *C*. *elegans* genome, but unlike *D*. *melanogaster*, the majority of the *C*. *elegans* balancers suppress recombination over only small chromosomal regions near their breakpoints. Inversions that suppress exchange, some with recessive lethal mutations and some with dominant visible markers, also exist in *Mus musculus*, but they are available for only a small portion of the genome [31,32].

## The hidden secrets of balancer chromosomes

Despite the pervasiveness of balancers in *D*. *melanogaster* research, the precise positions of the inversion breakpoints on the most commonly used balancers were only recently elucidated by whole-genome sequencing [33]. Forty-four of the 48 breakpoints on the most commonly used X, second, and third chromosome balancers were mapped using a combination of short-read Illumina sequencing and mate-pair sequencing. Subsequently, Ghavi-Helm and colleagues [34] used chromatin confirmation capture to estimate the positions of the four remaining breakpoints.

Although other balancers do exist in *D*. *melanogaster*, the balancers that have been sequenced are found in over 95% of the more than 40,000 stocks carrying at least one balancer at the Bloomington Drosophila Stock Center (https://bdsc.indiana.edu/stocks/stockdata.html). Furthermore, many of the inversions present on the balancers that were sequenced are also present on balancers that have not been sequenced.

Identifying the genomic position of each breakpoint yielded some surprising findings. Thirty-one breakpoints directly bisect a protein-coding gene, including the 65D breakpoint on *TM3* that bisects all transcripts of the highly conserved tumor suppressor gene *p53* [35]. Other genes have altered expression not because they are directly disrupted, but because of their proximity to a breakpoint. For example, the *light* gene, which encodes a cellular trafficking protein, is likely misexpressed on *SM5* due to abnormal juxtaposition of euchromatic and heterochromatic regions at the 40F inversion breakpoint [36]. Whole-genome sequencing also determined that 117 protein-coding genes are present in a large duplication carried by *SM5* (Fig 2).

Sequencing has shown that some balancers have been incorrectly labeled in stocks, and it is not difficult to see how that might happen. Most second chromosome balancers, for example, are marked with only one easy-to-identify dominant visible marker, *Curly*, leaving no simple way to determine if a stock carries *CyO*, *SM1*, *SM5*, or *SM6*. Indeed, in one study, out of 22 second chromosome balancers sequenced, four were mislabeled [36]. This should be a concern to researchers studying a mutant allele near a breakpoint or within a region poorly balanced by a particular balancer, such as the 42A to 58A segment of *SM1*. Fortunately, recessive markers can help distinguish balancers, and PCR primers are now available for 40 of the 44 breakpoints sequenced [35–37].

Another source of gene disruption on balancer chromosomes are SNPs and indels. Because each balancer was created one time and then distributed to the Drosophila community, any mutations on the original chromosome would be spread to all stocks containing that balancer. Because X-irradiation and ethyl methanesulfonate (EMS) mutagenesis were used to induce rearrangements and add visible markers, the number of mutations is probably higher on balancers. Indeed, sequencing a panel of balancers with common origins revealed many shared deleterious alleles, such as nonsense and splice-site variants. A panel of second chromosome balancers, for example, revealed 35 nonsense and 62 likely deleterious splice-site mutations shared among the all the stocks sequenced, as well as 8,898 missense variants whose impacts are unclear but may affect protein function [36]. Because balancers cannot easily replace deleterious alleles by crossing over, they are likely to accumulate unique mutations over time. One sequenced *SM5* balancer, for example, was found to have one nonsense and 24 missense variants that were not found on the other four *SM5* balancers sequenced [36].

In addition to the accumulation of SNPs and indels, sequencing revealed that balancers diverge in sequence as the result of rare double crossovers within inverted segments. For example, multiple X chromosome balancers had tracts of unique sequence within the 8.5-Mb *In(1)dl-49* inversion, which lies in the middle one-third of the *X* chromosome. These novel tracts were introduced from their structurally normal-sequence homologs. All of these double crossovers replaced a female-sterile allele of the *singed* gene with a normal allele, which may have provided the new balancers with a competitive advantage in stock populations. Although crossovers between effective balancers and their homologs are rare, gene conversions appear to occur at rates similar to or higher than normal [1], providing a mechanism by which shorter tracts of new sequence can be introduced.

## What balancers can teach us about crossover suppression by breakpoints

The distance over which inversion breakpoints suppress exchange is unknown and has been challenging to study using traditional marker-based approaches. Whole-genome sequencing of balancers has helped us chip away at this question. Because a large region at the distal end of left arm of *TM3* can be exchanged with normal-sequence homologs by single crossovers, historical recombination events have been preserved in this interval. Sequencing several *TM3* stocks revealed exchange events as close as 2 Mb from the distalmost inversion breakpoint, providing the first direct observation of the closest distance to a breakpoint a crossover can form in the face of crossover suppression [35].

In a subsequent study [1], crossovers were seen approximately 1 Mb from balancer breakpoints, but, interestingly, gene conversions were observed evenly distributed along the chromosomes and in close proximity to the breakpoints. This tells us that breakpoints have no effect on the placement of double-strand breaks or their repair into gene conversions. Considering data showing that gene conversions do not respond to interference or to the inhibition of meiotic recombination seen near centric heterochromatin, known as the “centromere effect” [33], it appears that inversion breakpoints may suppress exchange through a mechanism similar to interference or the centromere effect.

## The future of balancers

The coming years will likely see precise changes made to balancers using new tools, such as CRISPR and transgene technologies, either to make structural modifications to enhance their function or to introduce alternative markers. Indeed, GFP-expressing transgenes have been added to balancers to speed up screening in mutagenesis experiments by, for example, allowing for the easy identification of balancer heterozygotes that might be selected for or against [38–40]. Multiply rearranged chromosomes will likely be created for other species of *Drosophila*, allowing the maintenance of deleterious alleles and experiments involving complex crosses. Balancer chromosomes have a rich history and have been key to the development of *D*. *melanogaster* as a prominent model organism, and it is clear they will be essential to the *Drosophila* community for many years to come.

## References

[1] Crown NK, Miller DE, Sekelsky J, Hawley SR. Local inversion heterozygosity alters recombination throughout the genome. Curr Biol. 2018;28: 1 19. 10.1016/j.cub.2017.11.007 30174188 PMC6156927

[2] Stone W, Thomas I. Crossover and disjunctional properties of X chromosome inversions in *Drosophila melanogaster*. Genetica. 1935;17: 170 184. 10.1007/bf01984187

[3] Sturtevant A, Beadle G. The relations of inversions in the X chromosome of *Drosophila melanogaster* to crossing over and disjunction. Genetics. 1936;21: 554 604. 17246812 10.1093/genetics/21.5.554 PMC1208722

[4] Sturtevant A. A Case of rearrangement of genes in Drosophila. Proc National Acad Sci. 1921;7: 235–237. 10.1073/pnas.7.8.235 16576597 PMC1084859

[5] Sturtevant A. A third group of linked genes in *Drosophila ampelophila*. Science. 1913;37: 990 992. 10.1126/science.37.965.990 17833164

[6] Sturtevant A. A crossover reducer in *Drosophila melanogaster* due to inversion of a section of the third chromosome. Biologisches Zentralblatt. 1926; 697 702. 10.1111/j.1365-2818.1930.tb01489.x/abstract

[7] Muller H. Genetic variability, twin hybrids and constant hybrids, in a case of balanced lethal factors. Genetics. 1918;3: 422 499. 17245914 10.1093/genetics/3.5.422 PMC1200446

[8] Muller HJ. An Oenothera-like case in Drosophila. Proc National Acad Sci. 1917;3: 619–626. 10.1073/pnas.3.10.619 16586762 PMC1091337

[9] Muller H. The measurement of gene mutation rate in Drosophila, its high variability, and its dependence upon temperature. Genetics. 1928;13: 279 357. 17246553 10.1093/genetics/13.4.279 PMC1200984

[10] Muller H, Prokofyeva A. The individual gene in relation to the chromomere and the chromosome. Proc National Acad Sci. 1935;21: 16–26. 10.1073/pnas.21.1.16 16577650 PMC1076520

[11] Lewis E, Mislove R. New mutants report. Drosophila Information Service. 1953;27: 57 58.

[12] Schultz J, Redfield H. Interchromosomal effects on crossing over in Drosophila. Cold Spring Harbor symposia on quantitative biology. 1951;16: 175 197. 10.1101/sqb.1951.016.01.015 14942738

[13] Grell R, Lewis E. New mutants report. Drosophila Information Service. 1956;30: 71.

[14] Mislove R, Lewis E. New Mutants Report. Drosophila Information Service. 1954;28: 77.

[15] Merriam J. FM7: first multiple seven. Drosophila Information Service. 1968;43: 64.

[16] Ward L. The genetics of curly wing in Drosophila. Another case of balanced lethal factors. Genetics. 1923;8: 276 300. 17246014 10.1093/genetics/8.3.276 PMC1200750

[17] Mislove R, Lewis E. SM5: Second Multiple 5. Drosophila Information Service. 1955;29: 75.

[18] Oster I. A new crossing-over suppressor in chromosome 2 effective in the presence of heterologous inversions. Drosophila Information Service. 1956;30: 145.

[19] Craymer L. New Mutants Report. Drosophila Information Service. 1984;60: 234 236.

[20] Lewis E. New Mutants Report. Drosophila Information Service. 1960;34: 51.

[21] Lindsley D, Zimm G. The Genome of *Drosophila melanogaster*. San Diego: Academic Press; 1992.

[22] Hazelrigg T, Kaufman T. Revertants of dominant mutations associated with the antennapedia gene complex of *Drosophila melanogaster*: Cytology and Genetics. Genetics. 1983;105: 581 600. 17246168 10.1093/genetics/105.3.581 PMC1202175

[23] Ashburner M. New Mutants Report. Drosophila Information Service. 1972;49: 34.

[24] Schaeffer SW, Goetting-Minesky PM, Kovacevic M, Peoples JR, Graybill JL, Miller JM, et al Evolutionary genomics of inversions in *Drosophila pseudoobscura*: Evidence for epistasis. Proc National Acad Sci. 2003;100: 8319–8324. 10.1073/pnas.1432900100 12824467 PMC166227

[25] Edgely M. Genetic balancers Wormbook 2006; 10.1895/wormbook.1.89.1 PMC4781404 18050450

[26] Merritt BB, Cheung LS. GRIBCG: a software for selection of sgRNAs in the design of balancer chromosomes. Bmc Bioinformatics. 2019;20: 122 10.1186/s12859-019-2712-x 30866794 PMC6416924

[27] Schwartz HT, Sternberg PW. A toolkit of engineered recombinational balancers in *C*. *elegans*. Trends Genet. 2018;34: 253–255. 10.1016/j.tig.2018.01.009 29395380 PMC5878134

[28] Dejima K, Hori S, Iwata S, Suehiro Y, Yoshina S, Motohashi T, et al An aneuploidy-free and structurally defined balancer chromosome toolkit for *Caenorhabditis elegans*. Cell Reports. 2018;22: 232–241. 10.1016/j.celrep.2017.12.024 29298424

[29] Iwata S, Yoshina S, Suehiro Y, Hori S, Mitani S. Engineering new balancer chromosomes in *C. elegans* via CRISPR/Cas9. Sci Rep-uk. 2016;6: 33840 10.1038/srep33840 27650892 PMC5030659

[30] Chen X, Liao S, Huang X, Xu T, Feng X, Guang S. Targeted chromosomal rearrangements via a combinatorial use of CRISPR/Cas9 and Cre/LoxP technologies in *Caenorhabditis elegans*. G3 Genes Genomes Genetics. 2018;8: g3.2004732018. 10.1534/g3.118.200473 29950430 PMC6071600

[31] Hentges KE, Justice MJ. Checks and balancers: balancer chromosomes to facilitate genome annotation. Trends Genet. 2004;20: 252 259. 10.1016/j.tig.2004.04.004 15145578

[32] Ye Z, Sun L, Li R, Han M, Zhuang Y, Wu X, et al Generation of a mouse full-length balancer with versatile cassette-shuttling selection strategy. Int J Biol Sci. 2016;12: 911–916. 10.7150/ijbs.15172 27489495 PMC4971730

[33] Miller DE, Smith CB, Kazemi N, Cockrell AJ, Arvanitakis AV, Blumenstiel JP, et al Whole-genome analysis of individual meiotic events in *Drosophila melanogaster* reveals that noncrossover gene conversions are insensitive to interference and the centromere effect. Genetics. 2016;203: 159–171. 10.1534/genetics.115.186486 26944917 PMC4858771

[34] Ghavi-Helm Y, Jankowski A, Meiers S, Viales RR, Korbel JO, Furlong EE. Highly rearranged chromosomes reveal uncoupling between genome topology and gene expression. Nat Genet. 2019; 1–11. 10.1038/s41588-018-0328-0 31308546 PMC7116017

[35] Miller DE, Cook KR, Arvanitakis AV, Hawley SR. Third chromosome balancer inversions disrupt protein-coding genes and influence distal recombination events in *Drosophila melanogaster*. G3 Genes Genomes Genetics. 2016;6: 1959 1967. 10.1534/g3.116.029330 27172211 PMC4938649

[36] Miller DE, Cook KR, Hemenway EA, Fang V, Miller AL, Hales KG, et al The molecular and genetic characterization of second chromosome balancers in *Drosophila melanogaster*. G3 Genes Genomes Genetics. 2018;8: g3.2000212018. 10.1534/g3.118.200021 29420191 PMC5873907

[37] Miller DE, Cook KR, Kazemi N, Smith CB, Cockrell AJ, Hawley SR, et al Rare recombination events generate sequence diversity among balancer chromosomes in *Drosophila melanogaster*. Proc National Acad Sci. 2016;113: E1352–E1361. 10.1073/pnas.1601232113 26903656 PMC4790991

[38] Vef O, Cleppien D, Löffler T, Altenhein B, Technau GM. A new strategy for efficient in vivo screening of mutagenized Drosophila embryos. Dev Genes Evol. 2006;216: 105–108. 10.1007/s00427-005-0036-5 16328480

[39] Halfon MS, Gisselbrecht S, Lu J, Estrada B, Keshishian H, Michelson AM. New fluorescent protein reporters for use with the Drosophila gal4 expression system and for vital detection of balancer chromosomes. Genesis. 2002;34: 135–138. 10.1002/gene.10136 12324968

[40] Casso D, Ramírez-Weber F-A, Kornberg TB. GFP-tagged balancer chromosomes for *Drosophila melanogaster*. Mech Develop. 2000;91: 451–454. 10.1016/s0925-4773(00)00248-3 10704882

